# Symptomatic absorption of normal saline during transurethral resection of the prostate: a case report

**DOI:** 10.1186/s40981-022-00532-5

**Published:** 2022-06-21

**Authors:** Naomi Okuma, Hideki Hino, Madoka Kuroki, Tadashi Matsuura, Takashi Mori

**Affiliations:** Department of Anesthesiology, Osaka City University Graduate School of Medicine, 1-5-7 Asahimachi, Abeno-ku, Osaka, 545-8586 Japan

**Keywords:** Transurethral resection, Hyperchloremic metabolic acidosis, TUR syndrome

## Abstract

**Background:**

Transurethral resection of the prostate (TUR-P) could incidentally cause hyponatremia, known as TUR syndrome due to intravascular absorption of non-electrolytic irrigation fluid. Recently, normal saline has been used as an irrigation fluid in a new system named TURis (TUR in saline) to prevent TUR syndrome. However, rapid massive absorption of normal saline can also cause other systemic adverse events.

**Case presentation:**

A 71-year-old man underwent TURis for benign prostatic hyperplasia under spinal anesthesia. The patient lost consciousness which led upper airway obstruction and hypoxia 30 min after the surgery began. Blood gas test indicated hyperchloremic metabolic acidosis. While vasoactive agents were ineffective, the administration of bicarbonate significantly improved the symptoms and restored blood pressure.

**Conclusion:**

We experienced a case of hyperchloremic metabolic acidosis with decreased level of consciousness and hypotension during TURis. Administration of bicarbonate, but not phenylephrine, was effective for recovering blood pressure.

## Background

Transurethral resection of the prostate (TUR-P) can trigger hyponatremia, presenting as confusion, consciousness disturbance with or without hypotension, also known as TUR syndrome. TUR syndrome is induced by the rapid absorption of a substantial amount of irrigated non-electrolyte solution through the blood vessels of the prostate. TUR in saline (TURis) system is a relatively new method that utilizes normal saline as an irrigation fluid instead of a non-electrolyte solution [1]. It is true that TURis system is unlikely to induce hyponatremia, i.e., TUR syndrome, while it can cause systemic volume overload and various symptoms related to hyperchloremic metabolic acidosis. We describe a patient who developed symptomatic absorption of irrigated normal saline under spinal anesthesia. Written consent was obtained from the patient to present and publish this case report.

## Case presentation

A 71-year-old, 67-kg, 175-cm, otherwise healthy man was scheduled to undergo TURis for benign prostatic hyperplasia.

**Fig. 1:**
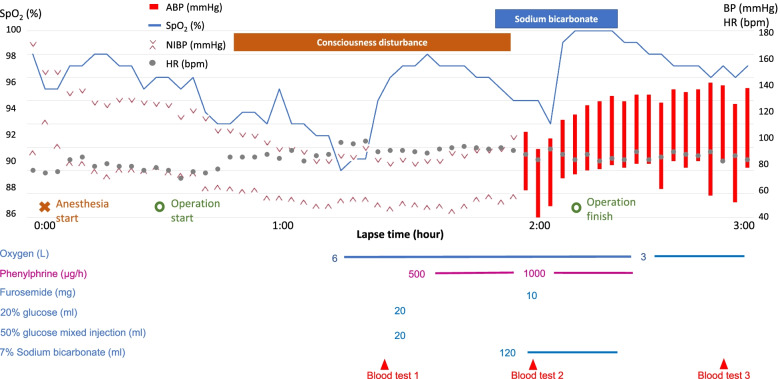
Anesthetic record of the surgery

No premedication was administered. Standard monitoring showed no abnormalities in vital signs except for high blood pressure (170/88 mmHg). Spinal anesthesia using 13 mg of 0.5% hyperbaric bupivacaine was administered. After the loss of cold sensation was reached to the level of T4, he was placed in the lithotomy position. Bicarbonate Ringer’s solution (BICANATE®, Otsuka Pharmaceutical Co., Ltd., Tokyo, Japan) containing 130 Na^+^, 4 K^+^, 2 Mg^2+^, 3 Ca^2+^, 109 Cl^−^, 28 HCO_3_^−^, and 4 citrate (mEq/L) was administered at the rate of 200 mL/h. TUR-P was performed using a bipolar high-frequency generator (Erbe VIO-3, AMCO Inc., Tokyo, Japan) and a 26-Fr continuous-flow bipolar resectoscope (OES Pro, Olympus Medical Systems Corp., Tokyo, Japan) under irrigation with normal saline that was hung approximately 100 cm above the patient. ECG showed ST-segment depression 30 min after the surgery began, and the patient lost consciousness and responded slightly to only strong stimuli such as pressure over the sternum. This was accompanied by upper airway obstruction and development of hypoxia, and oxygenation was initiated through a face mask. The patient was not intubated due to maintaining spontaneous breathing and acceptable hypoxia, keeping around 90% of SpO_2_ under the room air. Since venous blood gas sampling showed hypoglycemia (Table 1), a total of 14 g of glucose was administered. However, his consciousness did not recover. Approximately, at the same time, a gradual decrease in blood pressure was noted, which was not responsive to continuous phenylephrine administration (Fig. 1). The height of spinal anesthesia was comparable to that before the surgery. The rapid absorption of the irrigation solution and significant bleeding were suspected. The accumulated amount of normal saline irrigated during surgery was assumed to be up to 26 L. The venous blood test showed acidosis, hyperchloremia, and anemia as well (Table 1). After discussing the situation with the surgeons, we recommended reducing perfusion pressure and operation time. Total blood loss and urine output could not be measured because of the nature of surgery. Since hypotension and consciousness disturbance persisted after the operation, an arterial line was placed in the radial artery. Transthoracic echocardiogram (TTE) revealed no evidence of heart failure. Immediately after the administration of 120 mL of 8.4% sodium bicarbonate, the state of consciousness improved considerably, followed by restoration of blood pressure (134/57 mmHg) and normalization of the ST segment in ECG. After confirming an improvement in acidosis, 10 mg of furosemide was administered to ameliorate fluid overload. Chest X-ray imaging did not show significant congestion in the lung fields. The patient was transferred to the high care unit, and 560 mL of red blood cells were transfused. On postoperative day 4, the serum chloride level had improved to 108 mmol/L, and the patient was discharged.

**Table 1:** Blood gas test

	Sample 1 (venous)	Sample 2 (arterial)	Sample 3 (arterial)
pH	7.303	7.276	7.377
PaCO_2_, mmHg	37.0	34.9	36.4
PaO_2_, mmHg	57.2	82.9	62.9
Hb, g/dL	8.4	8.5	9.0
HCO_3_^−^, mmol/L	17.8	15.7	20.9
Na^+^, mmol/L	143	142	145
K^+^, mmol/L	3.0	3.0	3.1
Cl^−^, mmol/L	122	124	122
Glucose, mg/dL	66	108	108

## Discussion

The conventional TUR system, which involves patient’s body being in the energizing circuit with a non-electrolyte irrigation solution, occasionally causes hyponatremia, also known as TUR syndrome, due to the absorption of non-electrolyte irrigating solutions. In contrast, the TURis system is an alternative system that uses bipolar resection where the human body is electrically insulated from the energizing circuit and uses normal saline as the irrigating fluid. These properties contribute to the prevention of hyponatremia [1, 2].

However, some case reports show symptomatic absorption of irrigated normal saline during surgery. An abrupt large amount of normal saline has been reported to induce high chloride metabolic acidosis leading to various symptoms [3]. In another case report, the patient’s pH changed from 7.41 to 7.28 in 120 min due to rapid absorption of 6 L of irrigated normal saline [4]. The assumed amount of normal saline absorbed in our case was approximately 8.4 L, which was calculated based on a formula using serum chloride concentration in a previous report [5].

Metabolic acidosis can affect consciousness and hemodynamics. Although the acceptable pH level remains to be disputable, a pH around 7.1 or 7.2 seemed to be the borderline for unresponsiveness to catecholamines [6]. Rapid blood pressure recovery after the improvement of acidosis was due to the following reasons: Acidosis produces a substantial, reversible decrease in the number of cell surface α-adrenergic receptors and reduces the contractile force of the cardiac muscle. Arterial vasodilation caused by acidosis has been reported in the setting of in vitro experiments [7]. No deterioration of cardiac function and valvular regurgitation was found in TTE at the end of the surgery in our case; thus, the primary reason for hypotension might be vasodilation due to metabolic acidosis.

As a new method for TUR-P, holmium laser enucleation of the prostate (HoLEP) has been developed, which also uses normal saline as an irrigating fluid. The TURis system resects the tissue gradually; in contrast, the HoLEP system uses a laser to vaporize the tissue along with hemostasis. Therefore, the risk of bleeding and absorption of irrigating fluid is expected to be lower than that of the TURis system. However, normal saline absorption leading to hyperchloremic metabolic acidosis and volume overload in the HoLEP has also been reported [3, 8–10].

Gas emboli, which are products of electrosurgical vaporization, could also cause hemodynamic deterioration. Gas embolism may lead to symptoms similar to those associated with TUR syndrome. A randomized controlled trial of air embolism during hysteroscopic surgery under general anesthesia using bipolar or monopolar has been reported. Air was found in almost all cases with transesophageal echocardiography (TEE), and some patients showed ST-segment changes, blood pressure drop, and decreased Et.CO_2_. A large amount of air was observed, especially in patients with absorption exceeding 1 L. [11] Although gas embolism could not be ruled out because Et.CO_2_ decrease was not monitored, it would not be the pathological mechanism in the present case. For gas embolism being the primary cause, administration of bicarbonate solution would have worsened acidosis and its symptoms.

In conclusion, we report a case of hyperchloremic metabolic acidosis with decreased level of consciousness and hypotension during TURis. There are various treatments for benign prostatic hyperplasia. Regardless of the surgical procedure, abnormality caused by irrigation fluid absorption should be considered. In general, spinal anesthesia seems to be optimal in TUR, because patients’ consciousness can be assessed in cases of hyponatremia. Even though in the TURis system electrolyte disturbance can develop, spinal anesthesia may be a reasonable choice to enable the assessment of patients’ consciousness.

## Data Availability

Not applicable

## Abbreviations

TUR-P: Transurethral resection of the prostate
TURis: Transurethral resection in saline
TTE: Transthoracic echocardiogram
HoLEP: Holmium laser enucleation of the prostate
TEE: Transesophageal echocardiography
